# High prevalence and characterization of extended-spectrum ß-lactamase producing *Enterobacteriaceae* in Chadian hospitals

**DOI:** 10.1186/s12879-019-3838-1

**Published:** 2019-02-28

**Authors:** Oumar Ouchar Mahamat, Manon Lounnas, Mallorie Hide, Yann Dumont, Abelsalam Tidjani, Kadidja Kamougam, Madina Abderrahmane, Julio Benavides, Jérôme Solassol, Anne-Laure Bañuls, Hélène jean-Pierre, Christian Carrière, Sylvain Godreuil

**Affiliations:** 1 Centre Hospitalier Universitaire de Montpellier, Laboratoire de Bactériologie, Université de Montpellier, Montpellier, France; 2 UMR MIVEGEC IRD-CNRS-Université de Montpellier, Laboratory of Bacteriology CHU, Arnaud de Villeneuve 371 avenue du doyen Gaston Giraud, 34295 Montpellier Cedex 5, France; 3 Service de laboratoire, Hôpital de la Mère et de l’Enfant, N’Djaména, Chad; 4 Faculté de Médecine, Université de N’Djaména, N’Djaména, Chad; 5 Service de laboratoire, Hôpital Général des Références Nationale, N’Djaména, Chad; 6 Service de laboratoire, Hôpital de la Renaissance, N’Djaména, Chad; 7 Departamento de Ecología y Biodiversidad, Facultad de Ciencias de la Vida, Universidad Andrés Bello, Santiago, Chile; 8 Centre Hospitalier Universitaire de Montpellier, Département Bio-pathologie cellulaire et tissulaire des tumeurs, Université de Montpellier, Montpellier, France; 9 Laboraoire Mixte International DRISA, IRD, Montpellier, France

**Keywords:** ESBL, *Enterobacteriaceae*, Clinical samples, Prevalence, Chad

## Abstract

**Background:**

Extended-spectrum ß-lactamase-producing *Enterobacteriaceae* (ESBL-PE) represent a major problem in the management of nosocomial infections. However, ESBL-PE are not systematically monitored in African countries. The aim of this study was to determine ESBL-PE prevalence in patients from three hospitals in N’Djamena, the capital city of Chad, and to characterize the genetic origin of the observed resistance.

**Methods:**

From January to March 2017, 313 non-duplicate isolates were recovered from various clinical specimens obtained from 1713 patients in the three main hospitals of N’Djamena. Bacterial species were identified by matrix-assisted laser desorption ionization-time of flight mass spectrometry. Susceptibility to 28 antibiotics was tested using the disk diffusion method on Müller-Hinton agar, and ESBL production was confirmed with the double-disc synergy test. The most prevalent ESBL genes associated with the observed resistance were detected using multiplex PCR followed by double-stranded DNA sequencing.

**Results:**

Among the 313 isolates, 197 belonged to the *Enterobacteriaceae* family. The overall ESBL-PE prevalence was 47.72% (*n* = 94/197), with a higher rate among inpatients compared with outpatients (54.13% vs. 34.37%*).* ESBL-PE prevalence was highest in older patients (≥60 years of age). *E. coli* was the most common ESBL-producer organism (63.8%), followed by *K. pneumoniae* (21.2%). ESBL-PE were mainly found in urine samples (75%). The CTX-M-1 group was dominant (96.7% of the 94 ESBL-PE isolates, CTX-M-15 enzyme), followed by the CTX-M-9 group (4.1%). 86% of resistant isolates harbored more than one ESBL-encoding gene. ESBL production was also associated with the highest levels of resistance to non-β-lactam drugs.

**Conclusions:**

The prevalence of ESBL-PE harboring resistant genes encoding ESBLs of the CTX-M-1 group was high (48%) among clinical isolates of three main hospitals in Chad, suggesting an alarming spread of ESBL-PE among patients.

**Electronic supplementary material:**

The online version of this article (10.1186/s12879-019-3838-1) contains supplementary material, which is available to authorized users.

## Background

Extended-spectrum ß-lactamase-producing *Enterobacteriaceae* (ESBL-PE) represent a major problem in the management of nosocomial infections, resulting in prolonged hospital stays, increased hospital charges, and higher mortality and morbidity rates [1]. ESBLs confer resistance to many antibiotics, such as penicillins, cephalosporins and aztreonam, but not to cephamycins, moxalactam and carbapenems. *Klebsiella pneumoniae* and *Escherichia coli* are the main ESBL-producing organisms worldwide. Although at a lower frequency, these enzymes have also been detected in several other members of the *Enterobacteriaceae* family, such as *Enterobacter* spp., *Citrobacter* spp., *Proteus* spp. and *Morganella morganii*. [2–4]. Therefore, all these species can contribute to ESBL spread in hospital settings. Moreover, due to the coexistence of various modifying enzymes on the same plasmid, ESBL-PE often are resistant also to fluoroquinolones, aminoglycosides, trimethoprim sulfamethoxazole and tetracycline. Thus, ESBL-PE frequently display a multidrug resistance phenotype and are an important cause of treatment failure [5, 6].

ESBLs are encoded by different genes [7] inserted in genetic mobile elements, such as plasmids, that facilitate their spread between bacterial species. The most common ESBLs belong to the CTX-M, SHV and TEM families [8, 9]. The CTX-M family, particularly CTX-M-15, has emerged worldwide, and is now the most common ESBL type in hospitals and in the community [10]. Although ESBL-mediated bacterial resistance is recognized as an important health problem, limited data are currently available on ESBL-PE prevalence and molecular characterization in Sub-Saharan Africa. Particularly, to our knowledge, there is no study on ESBL-PE prevalence in clinical isolates in Chad.

The purpose of this study was to determine the prevalence and genetic characteristics of ESBL-PE in three main hospitals in Chad.

## Methods

### Setting

This study was conducted in the three main hospitals of Chad from January to March 2017. These three hospitals are located in N’Djamena, the capital city of Chad (1.5 million inhabitants) and are: (i) the National Reference General Hospital (HGRN), a university teaching hospital and one of the first national reference health facility. This hospital has 750 beds, with 8517 admissions and 50,896 outpatients in 2016; (ii) the Mother and Child Hospital (HME), a university teaching hospital and the reference mother-child hospital in Chad. It has a capacity of 261 beds (including an intensive care unit), with about 5000 admissions and 45,000 outpatients in 2016; and (iii) the Renaissance Hospital (HR), a tertiary healthcare facility designed to receive patients with complicated/chronic diseases from other healthcare centers. It has 250 beds and 8 intensive care unit beds. In 2016, 1457 inpatients were admitted among 23,909 consultations.

### Sample collection and identification

We analyzed 1713 consecutive clinical specimens (urine, surgical wound, pus, stool, sperm and blood samples) sent to the microbiology laboratory of each of these three hospitals (HME: *n* = 623, HGRN: *n* = 505, HR: *n* = 585). From these specimens, 313 non-duplicated and clinically significant bacterial isolates were obtained. Identification of the bacterial species was performed using biochemical tests and then confirmed by matrix-assisted laser desorption ionization-time of flight (MALDI-TOF) mass spectrometry (Bruker Daltonics, Bremen, Germany).

### Antimicrobial susceptibility testing and ESBL-production

Antimicrobial susceptibility testing was performed with the disk diffusion method on Müller-Hinton agar, as recommended by the European Committee on Antimicrobial Susceptibility Testing (EUCAST) guidelines and using the EUCAST clinical breakpoints (Version 7.1) (http://www.eucast.org/ clinical_breakpoints/). The following antibiotics were tested: amoxicillin, amoxicillin-clavulanic acid, ticarcillin, ticarcillin-clavulanic acid, piperacillin, piperacillin-tazobactam, temocilin, cephalexin, cefpodoxime, aztreonam, cefotaxime, ceftazidime, cefepime, cefoxitin, ertapenem, imipenem, gentamicin, tobramycin, nethilmycin, amikacin, trimethoprim + sulfamethoxazole, nalidixic acid, ofloxacin, ciprofloxacin, levofloxacin, tetracycline, chloramphenicol and fosfomycin. ESBL production was confirmed with the double-disk synergy method [11]. In the case of high-level production of cephalosporinase, the double-disk synergy test was performed using cloxacillin-supplemented medium (250 mg/L).

### Molecular characterization of ESBL and associated genes

DNA was extracted from one single colony of each isolate by incubation in a final volume of 100 μL of distilled water at 95 °C for 10 min followed by centrifugation. The presence of the *bla*_CTX-M_ (CTX-M group 1, 2, 8, 9 and 25), *bla*_TEM_, *bla*_SHV_ and _*bla*OXA-like_ genes was assessed using a multiplex PCR method following the protocol by Dallenne et al. 2012 [12]. Primers are listed in Table 1. The cycling conditions were: 95 °C for 10 min, followed by 30 cycles of denaturation at 95 °C for 40s, annealing at 55 °C for 40s, elongation at 72 °C for 1 min, and a final elongation step at 72 °C for 7 min. DNA samples from reference *bla*_CTX-M_, *bla*_TEM_, *bla*_SHV_ and *bla*_OXA-like_-positive strains were used as positive controls. The plasmid-mediated quinolone resistance (PMQR) gene (*qnr (A, B, C, D, S)*, *aac (6′)-Ib-cr, qepA* and *oqxAB*) and the aminoglycoside resistance-conferring 16S rRNA methylase genes (*armA, rmtB and rmtC*) were assessed using PCRs as previously described [13, 14]. PCR products were visualized after electrophoresis (100 V for 90 min) on 2% agarose gels containing ethidium bromide. A 100 bp DNA ladder (Promega, USA) was used as marker size. PCR products were sequenced bidirectionally on a 3100 ABI Prism Genetic Analyzer (Applied Biosystems). The sequencing data were analyzed online using the BLAST tool available at the National Center for Biotechnology Information web page (https://blast.ncbi.nlm.nih.gov/Blast.cgi).

**Table 1:** Primers used for the detection of β-lactamase-encoding genes

PCR	ß-lactamase genes	Primers	Nucleotide sequences	Amplicon size (bp)
Multiplex I	TEM including TEM-1 and TEM-2	MultiTSO-T_for MultiTSO-T_rev	CATTTCCGTGTCGCCCTTATTC CGTTCATCCATAGTTGCCTGAC	800
	SHV including SHV-1	MultiTSO-S_for MultiTSO-S_rev	AGCCGCTTGAGCAAATTAAAC ATCCCGCAGATAAATCACCAC	713
	OXA-1, OXA-4 and OXA-30	MultiTSO-O_for MultiTSO-O_re	GGCACCAGATTCAACTTTCAAG GACCCCAAGTTTCCTGTAAGTG	564
Multiplex II	CTX-M-1, CTX-M-3 and CTX-M-15	MultiCTXMGp1_for MultiCTXMGp1-2_rev	TTAGGAARTGTGCCGCTGYA CGATATCGTTGGTGGTRCCAT	688
	CTX-M-2	MultiCTXMGp2_for MultiCTXMGp1-2_re	CGTTAACGGCACGATGAC CGATATCGTTGGTGGTRCCAT	404
	CTX-M-9 and CTX-M14	MultiCTXMGp9_for MultiCTXMGp9_rev	TCAAGCCTGCCGATCTGGT TGATTCTCGCCGCTGAAG	561
CTX-M-8/25	CTX-M-8/25	CTX-Mg8/25_for CTX-Mg8/25_re	AACRCRCAGACGCTCTAC TCGAGCCGGAASGTGTYAT	326

### Statistics

Statistical analyses were performed using the Epi Info software, version 3.5.3 (Centers for Disease Control and Prevention, Atlanta, GA, USA). Differences in the proportion of ESBL-producers between patient groups were assessed using the Chi-square test, while associations between the presence of ESBL-encoding genes and categorical variables (sex, age and source of infection) were tested using multinomial logistic regressions. A *p* value < 0.05 was considered as statistically significant.

## Results

### Bacterial isolates

MALDI-TOF mass spectrometry analysis of the 313 clinically significant isolates showed that 197 were *Enterobacteriaceae*, whereas the other 116 isolates included Gram-positive cocci (*Enterococcus* spp., *Staphylococcus* spp. and *Streptococcus* spp) and Gram-negative bacilli (*Pseudomonas aeruginosa* and *Acinetobacter baumanii*). Among the 197 *Enterobacteriaceae* isolates*,* 134 were from inpatients’ and 63 from outpatients’ samples. *Enterobacteriaceae* isolates were recovered from urine (*n* = 143), pus (*n* = 44), blood (*n* = 7), stool (n = 1), wound (n = 1) and sperm (n = 1) samples. The age of these 197 patients ranged from 1 to 83 years, and 52.79% were men (Table 2). Raw data in Additional file 1.

**Table 2:** Characteristics of the patients infected by ESBL-PE and non-ESBL-PE

Variables	ESBL-PE	Non- ESBL-PE	Odds Ratio (95% CI)	*P*-value
	(*n* = 94)	(*n* = 103)		
Sex				0.071
Women (*n* = 93)	38	55	1	
Men (*n* = 104)	56	48	1.68 (0.95–2.95)	
Age group (years)				
<15 (*n* = 39)	12	27	1	
15 - <60 (*n* = 131)	63	68	1.97 (0.92–4.24)	0.08
≥ 60 (*n* = 27)	19	8	5.14 (1.76–15.03)	0.002
Hospital				
HME (*n* = 82)	34	48	1	
HR (*n* = 46)	22	24	1.29 (0.62–2.67)	0.48
HGRN (*n* = 69)	38	31	1.73 (0.90–3.30)	0.09
Patient type				
Outpatients (*n* = 64)	22	42	1	
Inpatients (*n* = 133)	72	61	2.25 (1.21–4.18)	0.01

### ESBL-PE prevalence

Among the 197 *Enterobacteriaceae* isolates, 94 (47.7%) were defined as presumable ESBL-PE on the basis of the antimicrobial susceptibility testing results. Molecular analysis confirmed that these 94 isolates carried ESBL-encoding genes. ESBL-PE prevalence was not significantly different in the three hospitals: 55% (38/69) at HGRN, 48% (22/46) at HR, and 41% (34/82) at HME (Table 2). The proportion of ESBL-PE isolates was higher in inpatients than outpatients (54.13% vs. 34.37%, *p* < 0.001), and in older patients (≥60 years of age) than in the other two age groups (OR = 5.14, 95% CI = 1.76–15.03, *p =* 0.002). Sex was not significantly associated with ESBL-PE presence (*p =* 0.071) (Table 2).

Among the 94 ESBL-PE, *E. coli* was the predominant species (*n* = 60, 63.83%), followed by *K. pneumoniae* (*n* = 20, 21.28%), *M. morganii* (*n* = 5, 5.32%), *Enterobacter cloacae* (*n* = 4, 4.26%), *Providencia rettgeri* (n = 2, 2.13%), *Proteus mirabilis* (*n* = 1, 1.06%), *Enterobacter aerogenes* (n = 1, 1.06%) and *Citrobacter koseri* (n = 1, 1.06%). Moreover, 70 of the 94 ESBL-PE isolates (74.47%) were from urine and 21 (22.34%) from pus samples (Table 3).

**Table 3:** Distribution of ESBL-PE isolates according to the *Enterobacteriaceae* species and sample type

Species	Sample					
	Urine	Pus	Blood	Wound	Sperm	Total (n%)
	n (%)	n (%)	n (%)	n (%)	n (%)	
*Escherichia coli*	45 (64.2)	12 (60.0)	1 (50.0)	1 (100.0)	1 (100.0)	60 (63.8)
*Klebsiella pneumonia*	15 (21.4)	5 (25.0)	0 (0)	0 (0)	0 (0)	20 (21.2)
*Morganella morganii*	4 (5.7)	1 (5.0)	0 (0)	0 (0)	0 (0)	5 (5.3)
*Enterobacter cloacae*	2 (2.8)	1 (5.0)	1 (50.0)	0 (0)	0 (0)	4 (4.2)
*Providencia rettgeri*	2 (2.8)	0 (0)	0 (0)	0 (0)	0 (0)	2 (2.1)
*Proteus mirabilis*	1 (1.4)	0 (0)	0 (0)	0 (0)	0 (0)	1 (1.0)
*Enterobacter aerogenes*	1 (1.4)	0 (0)	0 (0)	0 (0)	0 (0)	1 (1.0)
*Citrobacter koseri*	0 (0)	1 (5.0)	0 (0)	0 (0)	0 (0)	1 (1.0)
Total	70 (100.0)	20 (100.0)	2 (100.0)	1 (100.0)	1 (100.0)	94 (100.0)

### Resistance patterns in ESBL-producing and non-ESBL-producing *Enterobacteriaceae*

**Fig. 1:**
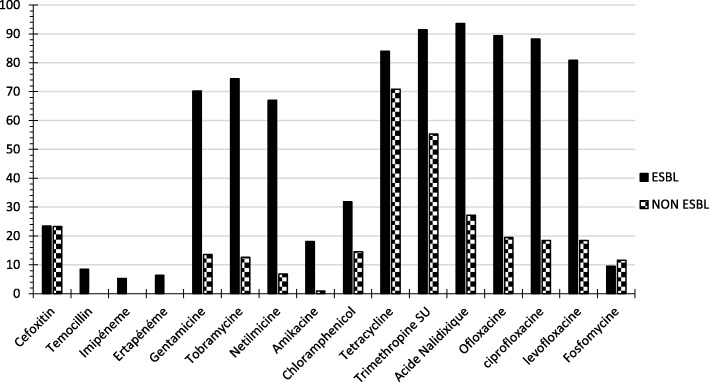
Antimicrobial resistance rates of ESBL-producing and non-ESBL-producing *Enterobacteriaceae* to other molecules

Resistance to antibiotics that are not hydrolyzed by ESBLs was more frequent in ESBL-PE than in non-ESBL-PE isolates, except for fosfomycin (Fig. 1). The rates of resistance to β-lactam antibiotics in ESBL-PE and non-ESBL-PE isolates were 93.62 and 27.18% for nalidixic acid, 89.36 and 19.42% for ofloxacin, 88.3 and 18.45% for ciprofloxacin, 80.85 and 18.45% for levofloxacin, 91.49 and 55.34% for sulfonamides, 74.47 and 12.62% for tobramycin, 70.21 and 13.59% for gentamicin, 67.02 and 6.8% for nethilmycin, and 18.09 and 0.97% for amikacin, respectively. Furthermore, the resistance rates of ESBL-PE isolates from inpatients and outpatients were 91.67 and 100.00% for nalidixic acid, 90.28 and 81.82% for ciprofloxacin, 73.61 and 59.09% for gentamicin, 15.28 and 27.27% for amikacin, and 80.56 and 95.46% for tetracycline. Therefore, the resistance rates in ESBL-PE and non-ESBL for cefoxitin, ertapenem and temocillin were 23,4% and 23,3%, 6,38% and 0,00%, 8,51 and 0,00%, respectively.

### Characterization of ESBL-encoding genes

The results of the PCR and sequencing analyses showed that the CTX-M group 1 was the most common (96.4% of isolates) ESBL type, and all the CTX-M-positive isolates carried the *bla*_CTX-M-15_ gene (Table 4). CTX-M group 9 was present in 4.1% of all ESBL-PEs (*bla*_CTX-M-27_ and *bla*_CTX-M-14_). The *bla*_CTX-M-27_ gene was detected only in *E. coli* isolates, and the *bla*_CTX-M-14_ gene only in *K. pneumoniae* isolates. The *bla*_SHV_*, bla*_CTX-M_ group 2, 8 and 25 genes were not detected in any of the ESBL-PE isolates. 86% of isolates carried more than one were associated with one to two other ß-lactamase genes (*bla*_TEM-1_ and *bla*_OXA-1_), 62% were associated with one to three PMQR (*qnrB, qnrD, qnrS, aac(6′)-ib-cr, oqxAB* and *qepA*) and 9% carried one to two 16S rRNA methylase genes (*armA, rmtB and rmtC*) Table 4. Two *E. coli* isolates harbored the *bla*_CTX-M-15_ gene in association with *bla*_CTX-M-27_, while one *K. pneumoniae* isolate carried the *bla*_CTX-M-14_ gene alone.

**Table 4:** Distribution of resistance genes in the 94 ESBL-producer isolates

Isolates (n)	Genes involved		
	β-lactamase genes	PMQR	16S rRNA methylase
*E. coli* (60)	CTX-M-15 (1)	*qnrS* (2)	*armA* (2)
	CTX-M-27 (3)	*oqxAB* (1)	*rmtC* (2)
	CTX-M-15/TEM-1 (14)	*qepA* (1)	*armA/rmtC* (1)
	CTX-M-9/TEM-1 (2)	*aac (6′)-Ib-cr* (24)	
	CTX-M-15/OXA-1 (17)	*qnrS/aac (6′)-Ib-cr* (4)	
	CTX-M-15/CTX-M-27/TEM-1 (2)	*qnrS/oqxAB* (1)	
	CTX-M-15/TEM-1/OXA-1 (19)	*qnrB/oqxAB* (1)	
	TEM-1 (1)	*qnrB/oqxAB/aac (6′)-Ib-cr* (2)	
	OXA-1 (1)	*oqxAB/aac (6′)-Ib-cr* (1)	
		*oqxAB/qnrS* (1)	
		*qepA/aac (6′)-Ib-cr* (1)	
*K. pneumonia* (20)	CTX-M-15 (4)	*oqxAB* (1)	*rmtB* (1)
	CTX-M-14 (1)	*qnrS/oqxAB/* (3)	*rmtC* (1)
	CTX-M-15/TEM-1 (5)	*qnrS/oqxAB/aac (6′)-Ib-cr* (1)	
	CTX-M-15/OXA-1 (2)	*qnrB*/*aac (6′)-Ib-cr* (1)	
	CTX-M-15/TEM-1/OXA-1 (8)	*qnrB/oqxAB* (1)	
		*qnrB/oqxAB/aac (6′)-Ib-cr* (5)	
		*oqxAB/aac (6′)-Ib-cr* (“)	
Other species (14)	CTX-M-15/TEM-1 (6)	*aac (6′)-Ib-cr* (2)	*rmtB* (1)
	CTX-M-15/OXA-1 (3)	*qnrB*/*aac (6′)-Ib-cr* (1)	*armA/rmtC* (1)
	CTX-M-15/TEM-1/OXA-1 (3)	*qnrD/aac (6′)-Ib-cr* (2)	
	OXA-1 (2)		

*n* number, *PMQR* plasmid-mediated quinolone resistance

## Discussion

The study reveals an ESBL-PE prevalence of 48% among clinical isolates in three major Chadian hospitals. Our results also confirm the spread of CTX-M-15 genes in isolates from African patients, and the finding that ESBL-PE display co-resistance to other antibiotic classes.

ESBL-PE prevalence varies widely between geographic areas. Low prevalence rates have been reported in Europe, USA and North America [15, 16], while high rates are usually observed in South America, Asia [17] and some African countries [18]. In Sub-Saharan Africa, and particularly in Central Africa, limited data are available on ESBL-PE. The prevalence found in our study (48%) is similar to the one reported for other African countries, such as Ghana (49.4%) [19], Gabon (50%) [20], Burkina Faso (58%) [21] and Cameroon 55.3% [22], and higher than in Nigeria (20.9%) [23] and Central African Republic (19.3%) [24]. Therefore, it confirms the spread of these bacteria in the African continent. A possible explanation for such high ESBL-PE prevalence is the high selective pressure generated by an important use of beta-lactam antibiotics in African countries, where they are frequently proposed as first-line treatment for bacterial infections caused by *Enterobacteriaceae* [25]*.* Other factors that contribute to their spread include non-prescription antimicrobial use, self-medication, poor hygiene, high burden of infectious diseases, consumption of counterfeit drugs, lack of antimicrobial resistance detection systems, and absence of diagnostic tools [26–28].

*E. coli* and *K. pneumoniae* were the most common ESBL-PE isolates and most of these isolates were from urine samples, in agreement with previous findings in India [29]. Urinary Tract Infection (UTI) is the most frequent bacterial infection worldwide in patients with nosocomial and community-acquired infections, and *Enterobacteriaceae* (mainly *E. coli* and *K. pneumoniae*) are generally the causal agent [30, 31].

ESBL-PE prevalence was significantly higher in isolates from inpatient than outpatient, as previously reported in Ghana and Rwanda [19, 31]. This pattern could be explained by the extensive use of ceftriaxone and cefotaxime as empirical antibiotic treatment in Chadian hospitals. Moreover, hospitalization has been identified as a high-risk factor for ESBL-PE infection, because ESBL-encoding genes are carried via plasmids that can be easily disseminated among the different bacteria that contaminate hospitalized patients [26, 32]. Both factors could be operating at the same time, and further research is needed to determine their contribution to the observed pattern. Like in previous studies, ESBL-PE were more frequent (*p* = 0.002) in isolates from older patients (≥60 years of age) [33]. This could be explained by the frequent administration of antibiotic therapy to older patient.

Regarding the association of resistance to different antibiotic classes, this study shows a positive association between ESBL-PE and resistance to quinolones, aminoglycosides (except amikacin), tetracycline, chloramphenicol and co-trimoxazole (trimethoprim/sulfamethoxazole), as previously reported in Burkina Faso and Gabon [21, 34]. The resistance to other antibiotic classes in ESBL-PE isolates is alarming, because it could further restrict the choice of adequate empirical therapy for the treatment of infections caused by these bacteria. In our study, isolates were susceptible to imipenem, ertapenem and amikacin. However, these drugs should be used with caution for empirical treatments in order to avoid emergence of carbapenem-resistant *Enterobacteriaceae*.

In our study, the most common resistance gene (*bla*_CTX-M-15_ in 96.7% of isolates) belonged to the CTX-M family. CTX-M-15 is now considered endemic in many countries and is rapidly disseminating among different *Enterobacteriaceae* species [14]. Similar to our study, high proportions of *bla*_CTX-M-15_-positive clinical isolates were reported in other Sub-Saharan African countries: Cameroon (96%) [22], Gabon (84.1%) [33], Burkina Faso (94%) [21], Ghana (98%) [32], and Nigeria (79%) [35].

The interpretation of the findings of this study is limited by the fact that there was no knowledge of the patients’ previous antibiotic treatments. Indeed, antibiotic treatments prior to sample collection could have favored the transient selection of resistant bacteria, and thus increased ESBL-PE prevalence compared with patients who were not previously treated with antibiotics.

## Conclusions

This report reveals a high ESBL-PE prevalence (48%) and the predominance of the CTX-M-15 enzyme among clinical isolates in three major Chadian hospitals. This emphasizes the urgent need to rationalize the use of antibiotics in hospital settings and to implement a national surveillance system for antibiotic-resistant bacteria to develop empirical treatment guidelines.

We also recommend further investigations to monitor carbapenem resistance and to determine whether healthy individuals act as ESBL-PE reservoirs in the community. These studies will contribute to better understand the mechanisms responsible for the spread of ESBL-PE in hospitals and communities.

## Additional file

Additional file 1: Raw data generated and analyzed during the current study. (XLSX 35 kb)

## Abbreviations

ESBL-PE: extended-spectrum ß-lactamase-producing Enterobacteriaceae
HGRN: National Reference General Hospital
HME: Mother and Child Hospital
HR: Renaissance Hospital
MALDI-TOF: matrix-assisted laser desorption ionization-time of flight
OR: Odd ratio
